# Ultrasound 2D strain measurement for arm lymphedema using deformable registration: A feasibility study

**DOI:** 10.1371/journal.pone.0181250

**Published:** 2017-08-30

**Authors:** Xiaofeng Yang, Mylin Torres, Stephanie Kirkpatrick, Walter J. Curran, Tian Liu

**Affiliations:** Radiation Oncology and Winship Cancer Institute, Emory University, Atlanta, GA, United States of America; North Shore Long Island Jewish Health System, UNITED STATES

## Abstract

**Purpose:**

Lymphedema, a swelling of the extremity, is a debilitating morbidity of cancer treatment. Current clinical evaluation of lymphedema is often based on medical history and physical examinations, which is subjective. In this paper, the authors report an objective, quantitative 2D strain imaging approach using a hybrid deformable registration to measure soft-tissue stiffness and assess the severity of lymphedema.

**Methods:**

The authors have developed a new 2D strain imaging method using registration of pre- and post-compression ultrasound B-mode images, which combines the statistical intensity- and structure-based similarity measures using normalized mutual information (NMI) metric and normalized sum-of-squared-differences (NSSD), with an affine-based global and B-spline-based local transformation model. This 2D strain method was tested through a series of experiments using elastography phantom under various pressures. Clinical feasibility was tested with four participants: two patients with arm lymphedema following breast-cancer radiotherapy and two healthy volunteers.

**Results:**

The phantom experiments have shown that the proposed registration-based strain method significantly increased the signal-to-noise and contrast-to-noise ratio under various pressures as compared with the commonly used cross-correlation-based elastography method. In the pilot study, the strain images were successfully generated for all participants. The averaged strain values of the lymphedema affected arms were much higher than those of the normal arms.

**Conclusions:**

The authors have developed a deformable registration-based 2D strain method for the evaluation of arm lymphedema. The initial findings are encouraging and a large clinical study is warranted to further evaluate this 2D ultrasound strain imaging technology.

## Introduction

Lymphedema, a swelling of the extremity, is a common long-term toxicity of cancer treatment. Lymphedema is the accumulation of lymph in the interstitial spaces, principally in the subcutaneous fatty tissues. For instance, arm lymphedema is a debilitating morbidity affecting approximately 25% of breast-cancer survivors [1–3]. Severe lymphedema can limit range of motion, cause pain or weakness, and may result in stiffness of the affected extremity. Currently, lymphedema is clinically diagnosed by medical history and physical examination that includes the circumferential or volume measurement of the affected arm [1–3]. Studies have shown a lack of consistency and rigor in these methods of measurement [4]. There is a clinical need for noninvasive and accurate diagnosis of lymphedema, particularly for early detection and accurate grading. If the lymphedema is diagnosed early (≤ Stage 1), successful intervention can reverse its development [5–7]. Moreover, many approaches have been attempted to treat lymphedema, including medications, physiotherapy and surgery. These treatments depend on the severity of the disease, which make accurate grading of lymphedema critical. This study’s objective is to develop a 2D strain technology to evaluate lymphedema. Lymphedema is marked by edema and chronic inflammation; therefore 2D strain value may be a parameter which can be used to characterize the severity of lymphedema.

Ultrasound (US) elastography is a technique for noninvasive characterization of tissue mechanical properties [8, 9]. Elasticity imaging, or strain imaging, describes the compressibility of biological tissues [9, 10]. In strain imaging, the displacement or deformation of tissue are estimated using pre- and post-compression image data [11, 12]. The axial strain is usually retrieved in two steps: displacement estimation, by matching pre-deformation RF data windows with post-deformation windows, and strain estimation, by differentiating the displacement field [13–16].

The quality of the elasticity image could be compromised by decorrelation between the pre- and post-compression ultrasound signals [17]. The main causes of this decorrelation are the changes of speckle patterns due to the complex scatter motion and out-of-plane motion of the probe [18]. Most elastography techniques estimate local displacements of tissue based on amplitude correlation [19, 20] or phase correlation of the radio-frequency (RF) signals [21, 22]. Cross-correlation (CC) method is commonly used estimator of distance (or similarity) beam between echo fields, and is capable of tracking small variations even when very low strains (less than 2%) are involved. However, elastograms remain degraded by decorrelation noise, especially when large and out of plane motion [23], non-uniformity of the ultrasound field and non-rigid tissue deformation [24] are present. In fact, incoherent motion and variations in the signal from scatters at high compression lead to displacement estimation errors [25] and ambiguities in the determination of the motion vectors [26]. Attempts have been made to adapt the correlation algorithm to provide subsample accuracy and estimation stability. These approaches proved to be valuable, however the displacement is estimated in sub-windows where it is supposed to be linear [27] or constant [23], and the continuity of the displacement field in the whole domain is not used. When the continuity assumption is violated, a tracking algorithm might not only fail to find the correct displacement at any particular point, but also propagate this incorrect estimate into other parts of the image, producing so-called drop-outs [28]. To guard against this, incorrect displacement estimates can be detected and replaced by values interpolated from nearby points, before they get a chance to propagate [29–31].

Another limiting factor of most current displacement tracking strategies is that the propagation direction is constrained to up–down, left–right or diagonal [28]. Most tissues exhibit anisotropic mechanical and functional properties, compressibility and viscoelastic behavior so that tissue motion and deformation are not limited to a single dimension [32, 33]. Hence, the necessity for measuring 2D and even 3D strain is evident. In this paper, we proposed a novel 2D strain imaging using registration of pre- and post-compression ultrasound B-mode images, which combines the statistical intensity- and structure-based similarity measures using normalized mutual information (NMI) metric and normalized sum-of-squared-differences (NSSD), with an affine-based global and B-spline-based local transformation model. The proposed method is not constrained to any particular set of directions, and is an alternative approach towards 2D or 3D strain estimation based on non-rigid registration of US images or volumes.

A part of this method was reported in SPIE Medical Imaging 2014 [34]. In this paper, we improved upon the previous intensity-based NMI similarity measurement and proposed a hybrid similarity measurement combined structure-based NSSD and intensity-based NMI to deal with the low contrast and signal-noise-rate (SNR) problem in ultrasound image. In addition, we expanded the phantom study. We showed 2D stain comparison and axial and lateral profile comparison through lesion for the CC-based and our registration-based method, and displayed 2D stain difference in 3D. We also investigated strain changes at different frames. And we also conducted initial clinical study to test its clinical feasibility.

## Methods

In registration-based strain imaging, the registration process is equivalent to finding the corresponding point before and after compression for each point in the ultrasound images, so it is similar as the displacement reconstruction in ultrasound elastography. For registration, an optimal deformation field is determined through spatially transforming an image (the post-pressure floating image) to achieve the best match between the deformed floating image and a second image (the pre-pressure reference image). In this work, a combined transformation model is used to parameterize the spatial transformation field. The global deformation of the tissue is modeled by an affine transformation, while the local tissue deformation is described by a B-spline transformation. Hybrid metric based on NMI and NSSD is used to combine the voxel- and structure-based similarity measure which is insensitive to the local intensity and contrast changes induced by pressure. Registration is achieved by minimizing a cost function, which represents a combination of the cost associated with the smoothness of the transformation and the cost associated with the image similarity.

**Fig 1:**
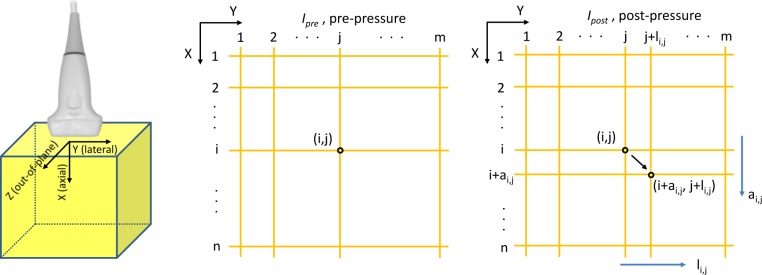
Axial, lateral and out-of-plane directions. The coordinate system is attached to the ultrasound probe. The sample (*i*,*j*) moved by (*a*_*i*,*j*_,*l*_*i*,*j*_). *a*_*i*,*j*_ and *l*_*i*,*j*_ are, respectively, axial and lateral displacements.

Let *I*_*pre*_ and *I*_*post*_ represent the two images acquired before and after compression (Fig 1). We will need to determine the two matrices *A* and *L* where the (*i*,*j*) component of *A*(*a*_*i*,*j*_) and *L*(*l*_*i*,*j*_) are the axial and lateral deformation of the pixel (*i*,*j*) of *I*_*pre*_. The axial and lateral strains are calculated by spatially differentiating *A* in the axial direction and *L* in the lateral direction, respectively. The two matrices *A* and *L* can be determined using deformable image registration assuming there is no the out-of-plane motion [17].

### Registration-based displacement estimation

#### Transformation model

The transformation field **T** maps each position in the reference pre-compression image *I*_*pre*_ to an anatomically corresponding location in the floating post-compression image *I*_*post*_. In the context of displacement estimation, the overall transformation **T**(*x*,*y*) combining a global and local transformation is applied here:

$$T(x,y)={Τ}_{global}(x,y)+T_{local}(x,y)$$ (1)

The first step to estimate tissue deformation under compression is to estimate the large overall motion, also called global displacement. For the global displacement, we used a rigid transformation model - 2D affine transformation, which is a linear transform, composed of the following geometric transformations: translation, rotation, shearing, and scaling. A 2D affine transformation **T**_*global*_(*x*,*y*) can be parameterized as:

$$T_{global}(x,y)=\left(\begin{array}{cc} u_{11} & u_{12}\\ u_{21} & u_{22} \end{array}\right)\left(\begin{array}{l} x\\ y \end{array}\right)+\left(\begin{array}{l} u_{13}\\ u_{23} \end{array}\right)$$ (2)

where the coefficients *U* parameterize the 6 degrees of freedom of the transformation.

The affine transformation captures only the global motion of deformation. An additional transformation is required, which models the local deformation of the tissue. For local displacement, we used a B-spline deformation model, which deforms an object by manipulating an underlying mesh of control points. The resulting deformation controls the shape of the object and produces a smooth and continuous transformation. The local transformation is defined as the B-spline weighted average of the control point *ϕ*_*ij*_,

$$T_{local}(x,y)=\sum_{i=0}^{3}\sum_{j=0}^{3}\beta_{i}(u)\beta_{j}(\nu )\phi_{i}{}_{j}$$ (3)

Here, the function *β*_*i*_ represents the *i*^*th*^ basis function of the B-spline *β*_0_(*u*) = (1−*u*)^3^/6, *β*_1_(*u*) = (3u^3^−6u^2^+4)/6, *β*_2_(*u*) = (−3u^3^+3u^2^+3u+1)/6, and β_3_(*u*) = *u*^3^/6, where 0 ≤ *u* ≤ 1. *ϕ*_*ij*_ = (*x*_*ij*_,*y*_*ij*_), *i*,*j* = 0,1,2,3 are the 4×4 control points that determine local transformation. In this paper, we used the smallest grid space *i* × *j* = 3 × 3 to achieve high resolution of the displacement [35].

#### Similarity measure

The similarity measure expresses the quality of the match between the transformed post-compression floating ultrasound image and the pre-compression reference image as a function of the transformation **T**. Since the local intensity and contrast of the ultrasound images could change after compression, we combined a voxel- and structure-based similarity measure. This hybrid metric is a combination of the popular normalized mutual information (NMI) metric and normalized sum-of-squared-differences (NSSD) metric:

$$\begin{array}{l} E_{similarity}(I_{pre},I_{post})=E_{NMI}(I_{pre},I_{post})-E_{NSSD}(I_{pre},I_{post})\\ \;=\frac{H(I_{pre})+H(I_{post})}{H(I_{pre},I_{post})}-\frac{1}{N}\sum_{}\left\|\frac{I_{pre}-u_{I_{pre}}}{\sigma_{I_{pre}}}-\frac{I_{post}-u_{I_{post}}}{\sigma_{I_{post}}}\right\| \end{array}$$ (4)

where *I*_*pre*_ denotes the pre-compression reference and *I*_*post*_ denotes the post-compression floating image; *H*(*I*_*pre*_) and *H*(*I*_*post*_) denote the marginal entropies *I*_*pre*_ and *I*_*post*_. The *H*(*I*_*pre*_,*I*_*post*_) denotes the joint entropy, which is calculated from the joint histogram of *I*_*pre*_ and *I*_*post*_. $\mu_{I_{pre}}=G_{s}*I_{pre}$ denotes the local intensity mean. $\sigma_{I_{pre}}=G_{s}*{\left(I_{pre}-\mu_{I_{pre}}\right)}^{2}$ denotes the local intensity variation of image *I*_*pre*_. The same denotations are used for *I*_*post*_. *G*_*s*_ denotes a Gaussian filter with kernel size *s*. In order to improve the robust of registration algorithm, we selected the kernel size *s* of Gaussian filter as 3 times the image voxel size of the displacement because it is usually selected as 2–3 times the image voxel size for NSSD to get more accurate local intensity and variation [36]. This hybrid similarity measure provides a better image alignment than using the NMI metric alone since the NSSD-term is an edge-based alignment metric, and it is not sensitive to the local image contrast changes [36].

#### Smoothness constraint

The B-spline interpolation of the displacement vectors imposes a certain degree of smoothness on the deformation field. However, this inherent smoothness is not controllable and is not always sufficient in preventing nonphysical deformations such as folding or large local stretching or shrinking. Therefore, a penalty term is used to constrain the deformation field, which is based on the bending energy of a thin plate of metal that is subjected to bending deformations [37]. The penalty term is composed of second-order derivatives of the deformation:

$$E_{smooth}(T)=\frac{1}{\Omega }\int_{0}^{X}\int_{0}^{Y}[(\frac{\partial^{2}T}{\partial x^{2}}{)}^{2}+{\left(\frac{\partial^{2}T}{\partial y^{2}}\right)}^{2}+2(\frac{\partial^{2}T}{\partial x\partial y})]dxdy$$ (5)

where Ω denotes the volume of the image domain. This quantity is the 2D equivalent of the bending energy of a thin-plate of metal and defines a cost function which is associated with the smoothness of the transformation. The regularization term penalizes only non-affine transformations.

#### Optimization

To find the optimal transformation, we minimize a cost function associated with the global transformation parameters *U*, as well as the local transformation parameters Φ. The cost function comprises two competing goals. A user-defined weighting factor *τ*(0 < *τ* < 1) controls the relative influence of *E*_*similarity*_ and *E*_*smooth*_, combining both into the overall cost function *E*_*total*_ as follows:

$$E_{total}(U,\Phi )=(\tau -1)E_{similarity}(I_{pre},T(I_{post}))+\tau E_{smooth}(T)$$ (6)

Here, *τ* is the weighting parameter, which defines the tradeoff between the alignment of the two images and the smoothness of the transformation. We used *τ* = 0.01 that it has been proven such parameter was best to optimize the joint cost function [35, 36]. Finding the parameters of the nonrigid transformation that optimize the joint cost function *E*_*total*_ requires an efficient and robust optimization algorithm. The optimization proceeds include two stages. During the first stage, the affine transformation parameters *U* are optimized using an iterative multi-resolution search strategy [38]. Since the smoothness term of the cost function is zero for any affine transformation, this step is equivalent to maximizing the image similarity measure. During the subsequent stage, the nonrigid transformation parameters Φ are optimized as a function of the cost function in Eq (6). In each stage we are using an iterative gradient descent technique, which steps in the direction of the gradient vector with a certain step size [39]. The gradient can be estimated very efficiently due to the local effect of the control point positions. This procedure is repeated until the cost function cannot be improved any further. We aim to improve the robustness and efficiency of the algorithm by employing a multi-resolution approach, starting with a coarse spacing that is successively refined using a B-spline subdivision algorithm [35].

### Strain calculation

Strain is defined as the deformation of an object, normalized to its original shape.[37, 40] In this study, the deformation of the object is caused by external compression and the relaxation of the tissue. A linear array transducer was used, and the ultrasound beam direction was taken as the *x*-axis, while the *y*-axis denoted the orthogonal direction within the imaging plane (Fig 1). The point in space is observed using an ultrasound transducer. The medium at this point undergoes an actual displacement, specified by vector $\bar{d}$. The displacement vector $\bar{d}$ contains two orthogonal components *d*_*x*_ and *d*_*y*_. The strains are obtained from the gradient of the displacement at that point.

## Experiments and results

### Phantom study

An elastography phantom (CIRS Model 059) was used to test the performance of the proposed registration-based strain technology. A dense mass (8 mm in diameter) inside the phantom was scanned and an elastic modulus of this the mass was 20 kPa ± 5kPa, which is at least two times greater than the elasticity of the background. The ultrasound scan was carried out with a clinical scanner (SonixTouch, Ultrasonix, British Columbia, Canada) with a linear array transducer (L14-5/38). The scan setting was the following: 10 MHz center frequency, 2 cm focal length, 4 cm depth, 72% gain, and 80-dB dynamic range. The pixel size of B-mode image is 0.10×0.10 mm^2^. The B-mode images and RF data of the multiple frames were acquired simultaneously with a uniform compression applied to the phantom surface by the probe.

We compared the proposed registration-based method with the traditional CC-based elastography method. We calculated the contrast to noise ratio (CNR) and signal-to-noise ratio (SNR) to assess the performance of our method, and performed quantitative comparison. The CNR and SNR are defined as:

$$CNR=\frac{Contrast}{Noise}=\sqrt{\frac{2{\left(m_{b}-m_{t}\right)}^{2}}{\sigma_{b}^{2}+\sigma_{t}^{2}}}\;\mathrm{and}\;SNR=\frac{Signal}{Noise}=\frac{m}{\sigma }$$ (7)

where *m*_*t*_ and *m*_*b*_ are the averaged spatial strain of the target and background, $\sigma_{b}^{2}$ and $\sigma_{t}^{2}$ are the strain variance of the target and background, and *m* and *σ* are the spatial average and variance of a window in the strain image, respectively.

**Fig 2:**
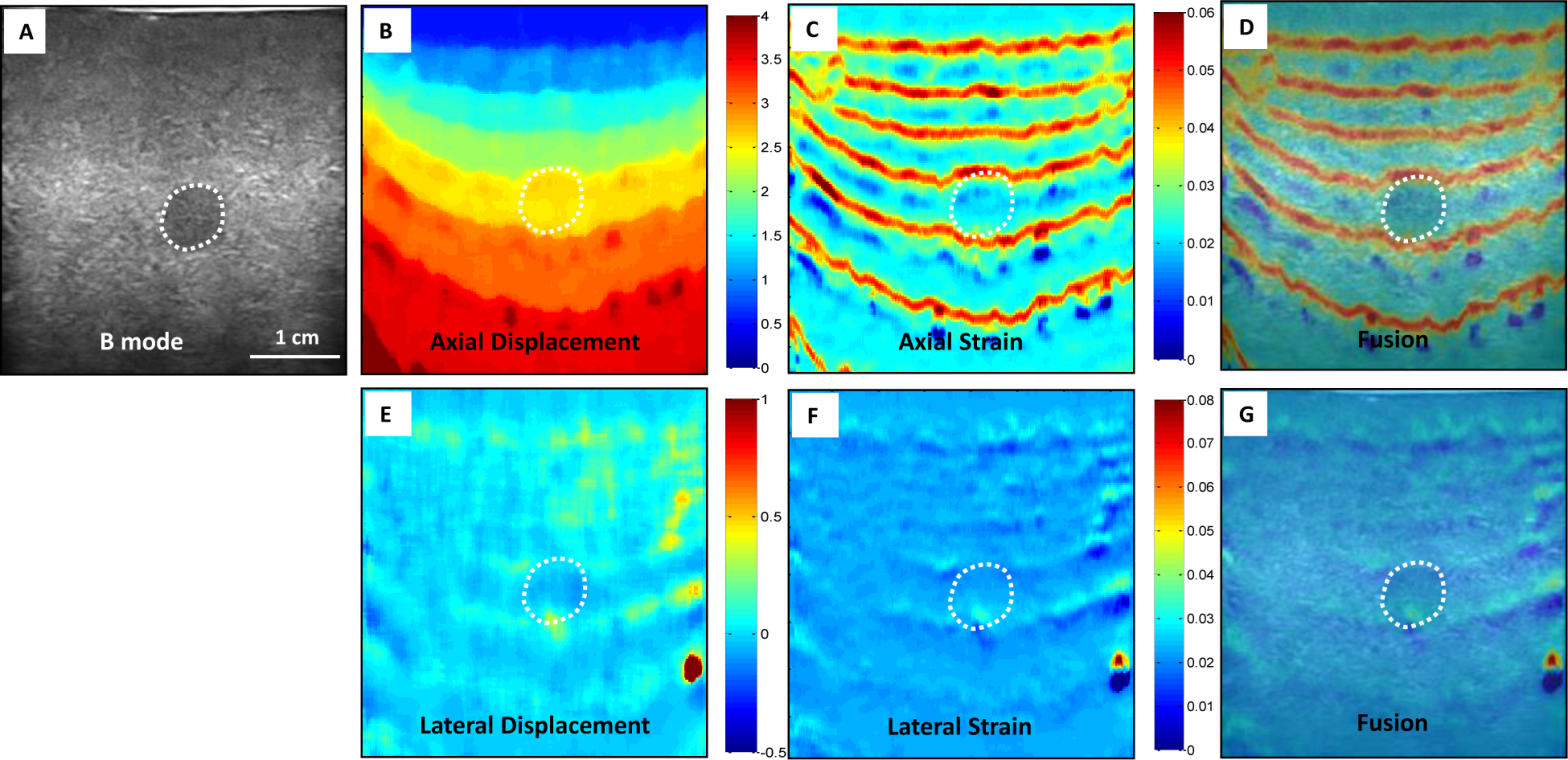
Phantom experiment results–CC-based method. (A) B-mode image of the elastography phantom; CC-based elastography results (B) axial displacement, (C) axial strain, and (D) axial strain and B-mode fused image; (E) lateral displacement, (F) lateral strain (G) lateral strain and B-mode fused image.

**Fig 3:**
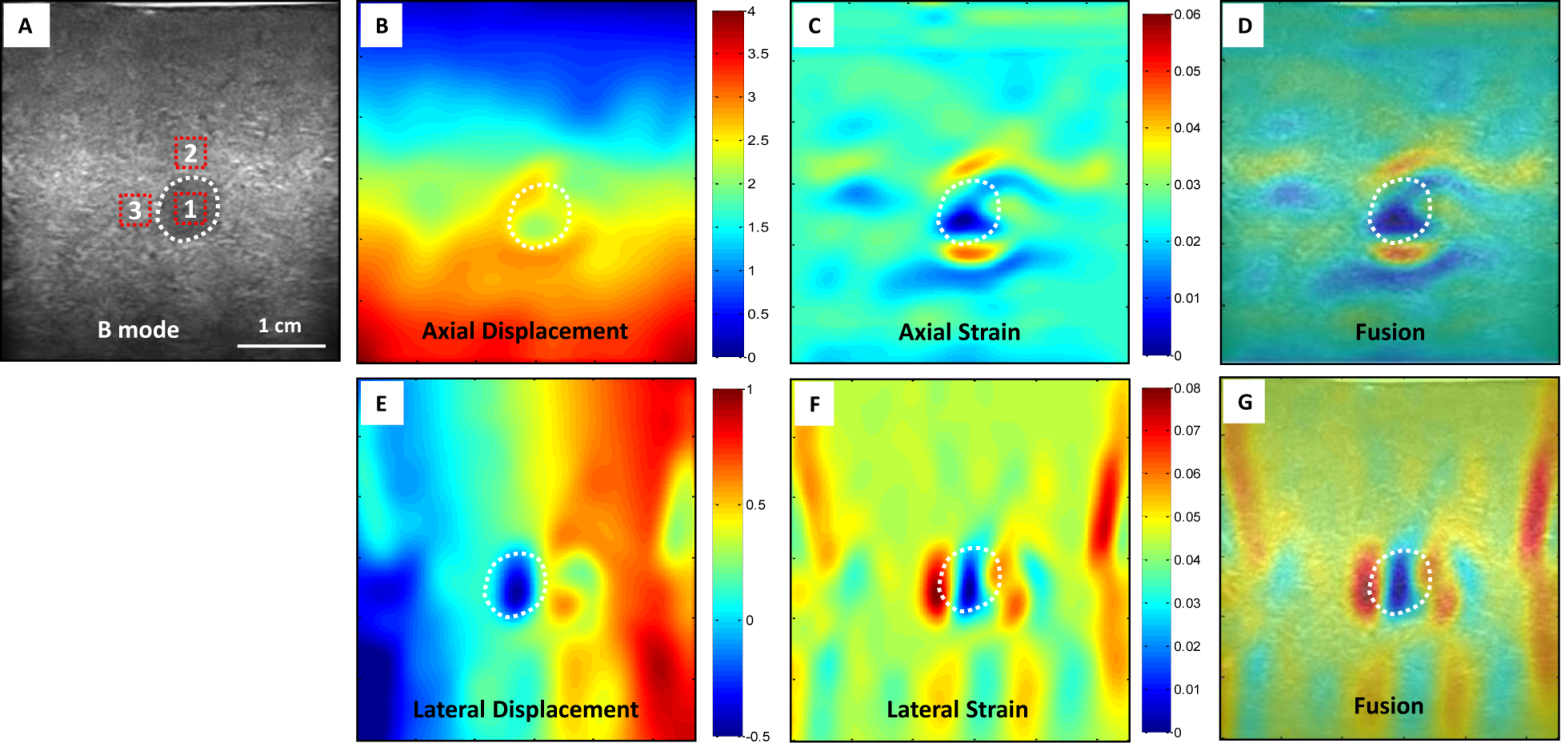
Phantom experiment results–Our method. (A) B-mode image of the elastography phantom; our registration-based elastography results (B) axial displacement, (C) axial strain, and (D) axial strain and B-mode fused image; (E) lateral displacement, (F) lateral strain (G) lateral strain and B-mode fused image.

Fig 2 shows the CC-based strain image results from the elastography phantom. We can see the block-match method based on the CC has many artifacts in axial strain image, and the lesion in the phantom appears almost invisible in lateral strain images. Fig 3 displays our registration-based strain image results. In this image, our proposed method can detect the lesion in both the axial and lateral strain images. In the displacement figures, the unit is pixel and red indicates larger deformation. In the strain figures, red indicates softer tissue with higher strain value.

**Fig 4:**
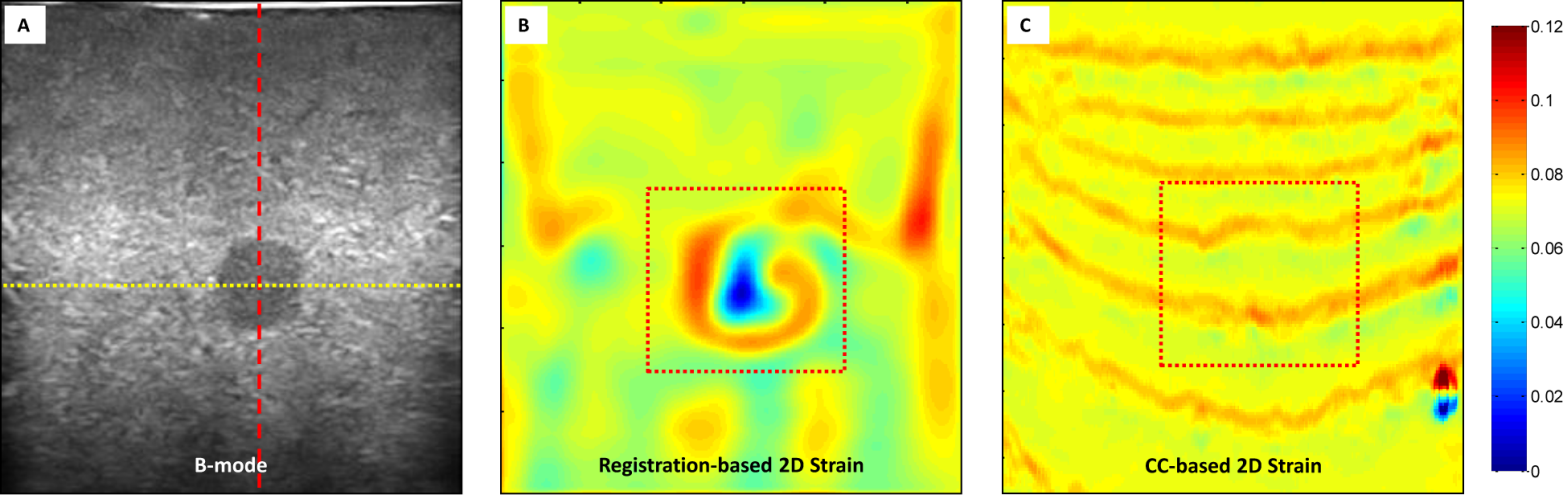
Phantom experiment results 2D strain comparison. (A) B-mode image of the elastography phantom; our registration-based elastography result (B) 2D strain image, and the CC-based elastography result (C) 2D strain image.

**Fig 5:**
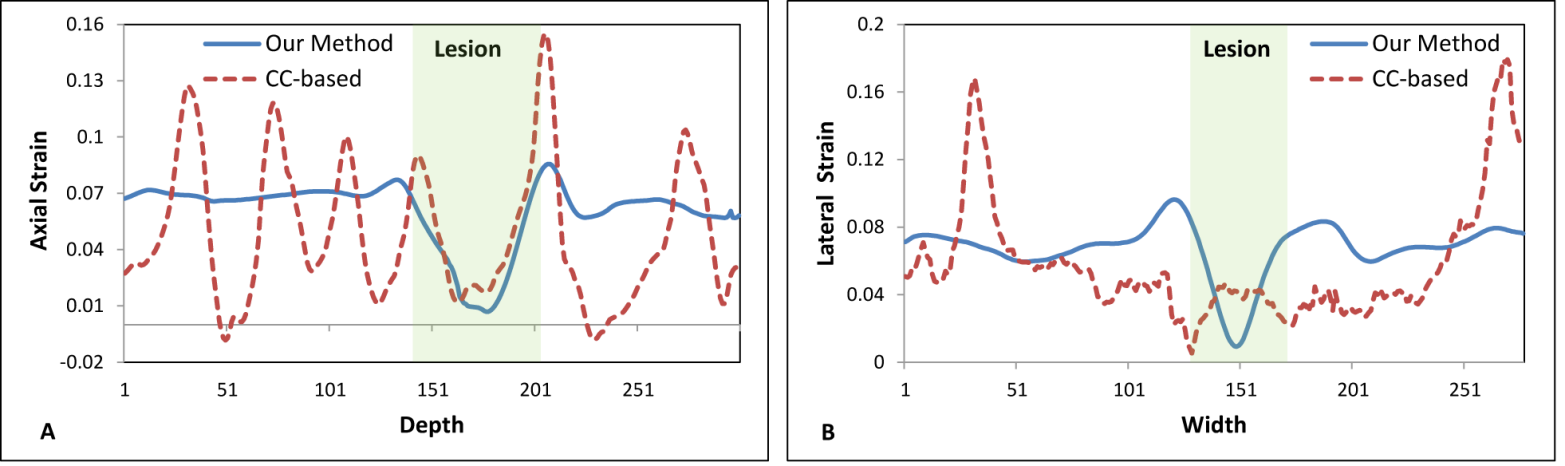
Axial and lateral profile comparison through the lesion. (A) Profile comparison of two methods through axial red dashed line in Fig 4 (A); (B) profile comparison of two methods through lateral yellow dotted line in Fig 4 (A).

**Fig 6:**
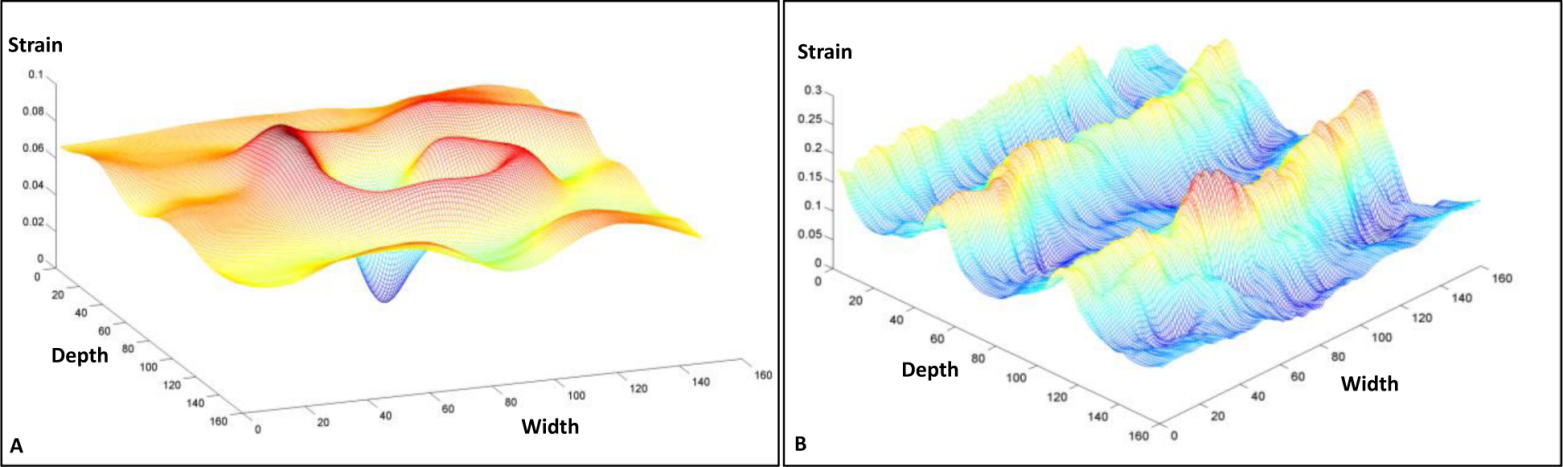
3D comparison of ROIs in strain images for the two methods. (A) Strain visualization of ROI in Fig 4 (B) for our registration-based method; (B) strain visualization of ROI in Fig 4 (C) for CC-based method.

Fig 4 illustrates the 2D strain image comparison of the registration-based and CC-based methods. The 2D strain images were calculated by pixel-by-pixel summing the axial and lateral strain images. Fig 5 shows the profile comparison through the lesion region at the axial and lateral directions as shown in Fig 4(A). The shadowing regions in this figure show the corresponding axial and lateral directions of the lesion region. The lesion in the phantom has a high stiffness and should have a lower strain value than the background. This point can be clearly seen for both methods in Fig 5(B). In the background, the CC-based method has heavily oscillated artifacts, while the background almost remains the constant strain value in our registration-based method, which is consistent with the fact that backgrounds composed of the same material should have a constant strain value. Comparing the two profiles in Fig 5(C), the lesion in CC-based method has almost the same lateral strain value with the background, and cannot be separated from the background, while the lesion in our registration-based method has a drastically lower strain value than the background, and can be easily detected from the background. Fig 6 further demonstrates the 3D visual strain comparison of the two regions of interest (ROIs) as shown in Figs 4(B) and (C) 2D strain images.

**Fig 7:**
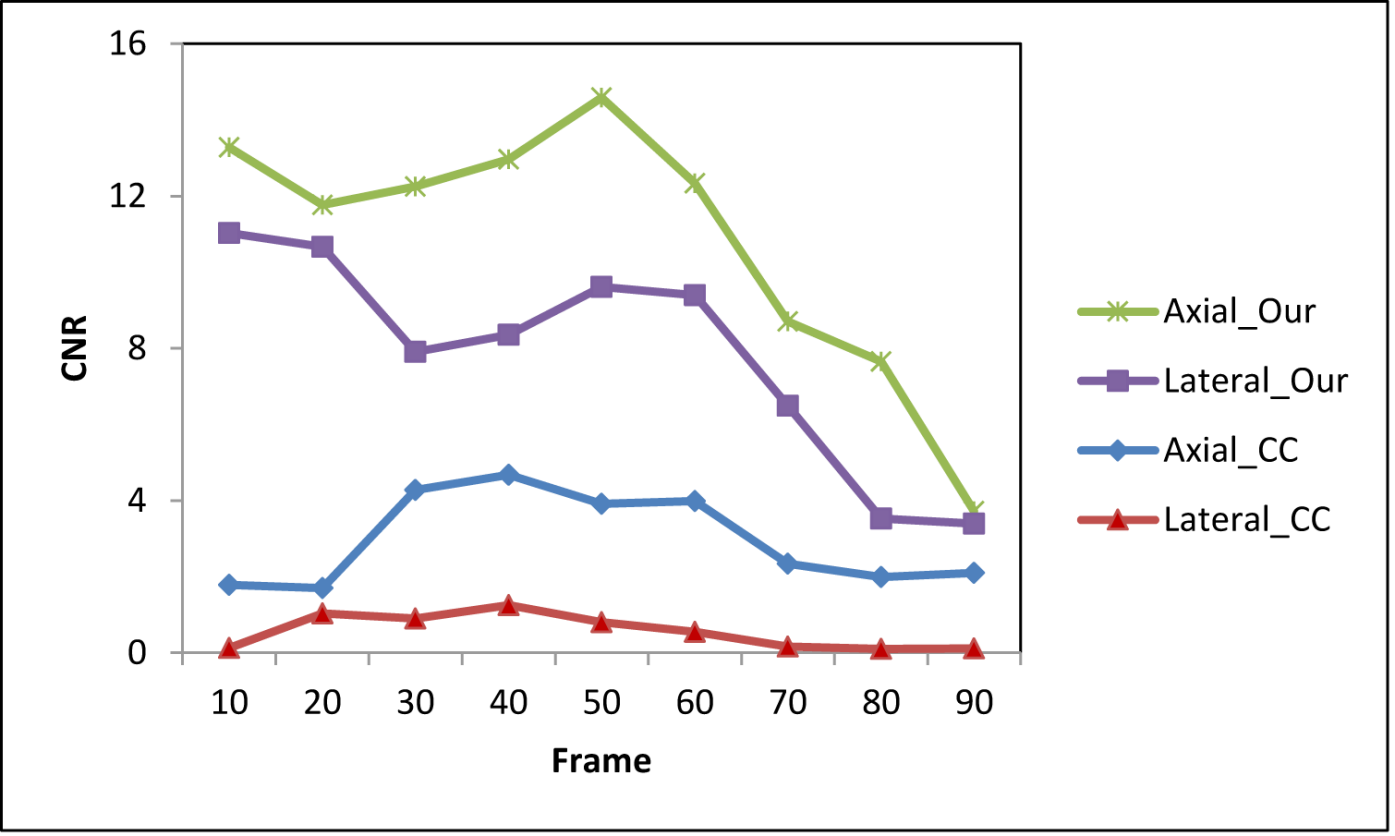
A comparison of the CNR between the two methods under different strains. This is the axial and lateral CNR from the two methods. The ROI 1 in Fig 3 was seen as a target and ROI 2 was viewed as the background. ROI 1 and 2 were used to calculate the CNR in the axial strain images of both methods, while ROI 1 and 3 were used to calculate CNR in lateral strain images of both methods.

**Fig 8:**
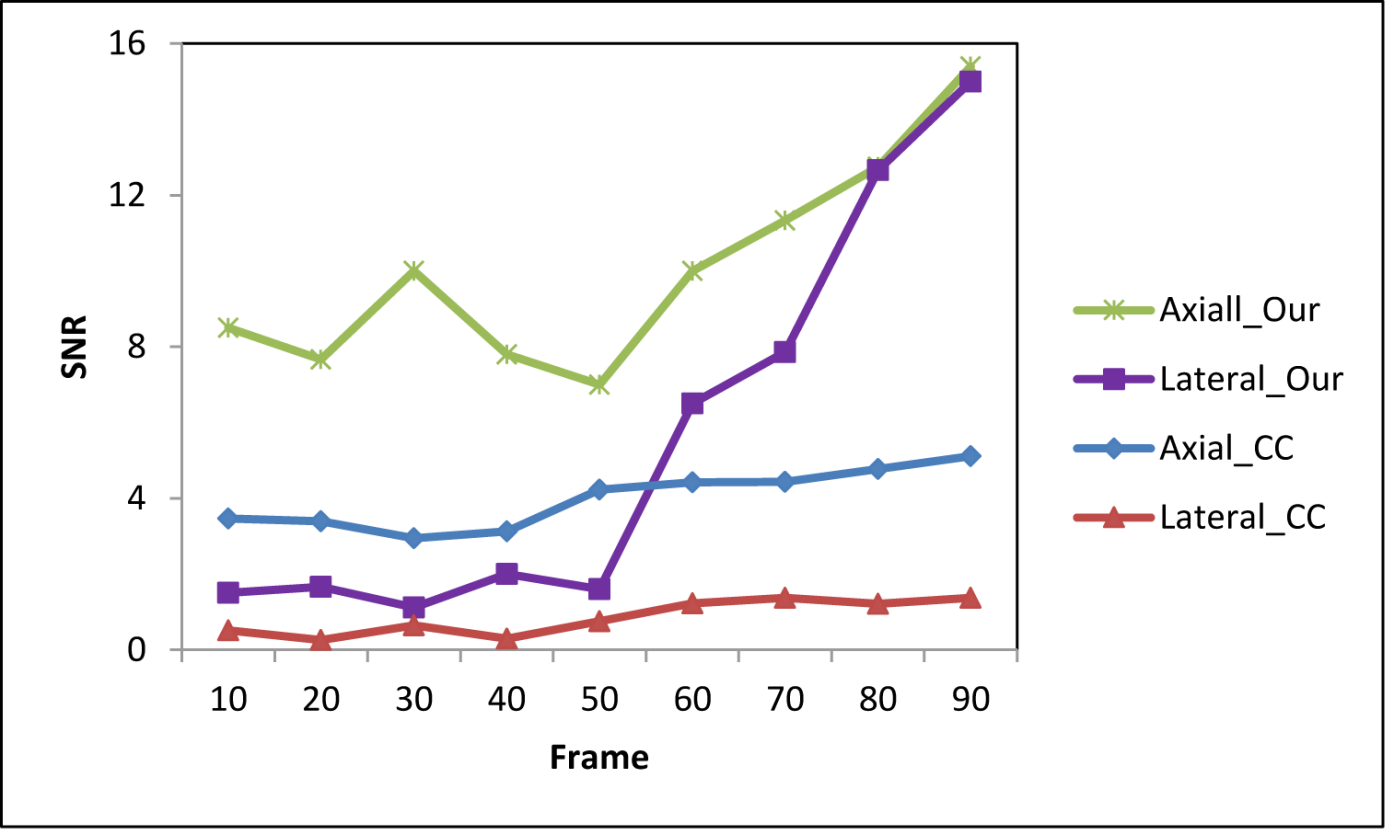
A comparison of SNR between the two methods under different strains. This is the SNR calculated from the axial and lateral strain images obtained from the two methods. The ROI 1 in Fig 3 was seen as a target and ROI 1 was used to calculate the SNR of both methods in both directions.

**Fig 9:**
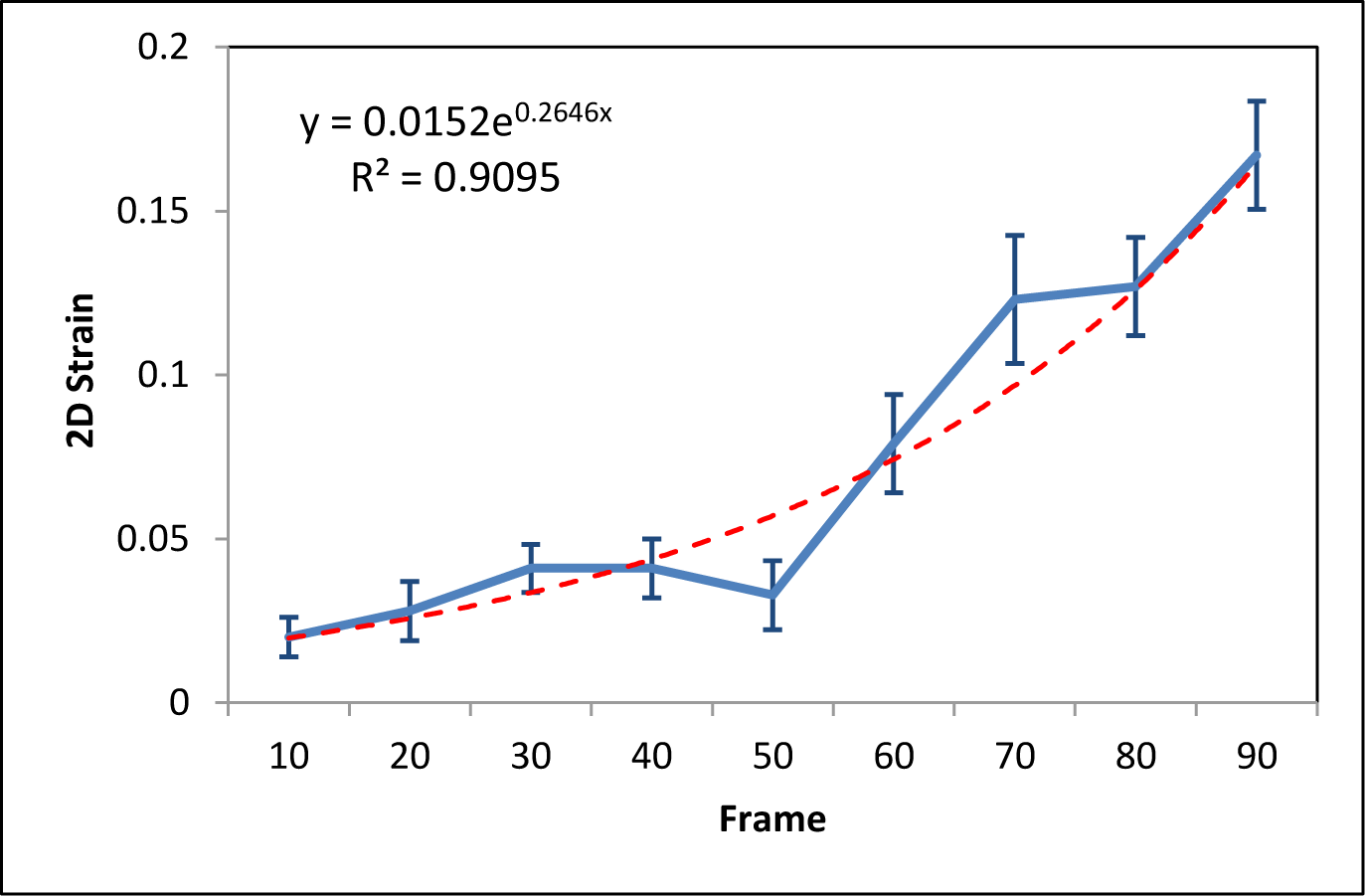
Strain curve at different frames (pressure) and the corresponding exponential fitting curve.

The CNR and SNR (Eq (7)) are used to quantitatively compare the registration-based and the CC-based strain methods. Ninety frames of B-mode images and RF data were acquired under probe-induced compression. The first frame was used as the reference frame, and every 10th frame (10th, 20th 30th, 40th, 50th, 60th, 70th, 80th and 90th) was used as the floating frames to compare the CNR and SNR under various pressures. Figs 7 and 8 show the CNR and SNR in the axial and lateral strains under various pressure of the two methods. While the CNR and SNR in the axial strain are higher than the lateral strain for both methods, the CNR and SNR of our method are higher than the CC-based method. For both axial and lateral strains, the CNRs increased with pressure at lower stress and decreased with pressure at higher stress, while the SNRs increased with pressure. Fig 9 shows the 2D strain curve of the lesion at various pressures. The average lesion strain increased with pressure. In this elastography phantom, the stiffness of the lesion was greater than the background. When we started to compress the phantom, the background showed greater shrinkage than the lesion. With the increase of pressure, the background shrank to some limit around the 50^th^ frame, so that it became difficult to shrink (e.g. strain hardening) under the increased compression. And then the lesion became “soft” as the compressed background and displayed similar strain values as the compressed background. So the lesion cannot be easily separated from the background with the increase of pressure, which is demonstrated though the decreasing axial and lateral CNRs as shown in Fig 7. The average strain value of the lesion remained low and constant during the first 50 frames and then increased greatly after the 50^th^ frame as shown in Fig 9, which is consistent with our above analysis. The increased 2D strain curve closely conformed to an exponential curve. The *R*^*2*^ = 0.91 demonstrated excellent curve fitting.

### Clinical study

We conducted a pilot study to test clinical feasibility of the registration-based 2D strain method under Emory Institutional Review Board approval. The inclusion criteria are 1) healthy volunteers over age 18 who serve as the control group, and 2) patients over age 18 who have developed arm lymphedema after breast surgery or radiotherapy. We enrolled 4 participants in this feasibility study: 2 healthy women (age: 38 and 48) and two patients (age: 50 and 52) who had developed arm lymphedema post breast-cancer radiotherapy (all participants have already signed the informed consent forms) between January, 2012 and December, 2013. Lymphedema clinical evaluation of these two patients was conducted by a certified lymphedema specialist with over 10-year experiences (S.K., DPT). The severity of lymphedema was graded on a 4-point system ranging from normal to severe lymphedema using CTC v. 2.0 lymphedema grade [41]. We designed an ultrasound elastography device that incorporates an ultrasound probe with an inflatable cuff attached to a manometer. The inflatable cuff provides an adjustable low pressure to compress the arm. This system provided accurate pressure control and reduce out-of-plane motion of the ultrasound probe. The subjects were scanned in the supine position and asked to remain silent during each acquisition, when the transducer was placed over the biceps. The *in-vivo* arm data was acquired using BPL9-5/55 probe with 256 elements under the following settings: 6.6 MHz center frequency, 2 cm focal length, 4 cm depth, 50% gain, and 80-dB dynamic range. The pixel size of B-mode image is 0.12×0.12 mm^2^.

**Fig 10:**
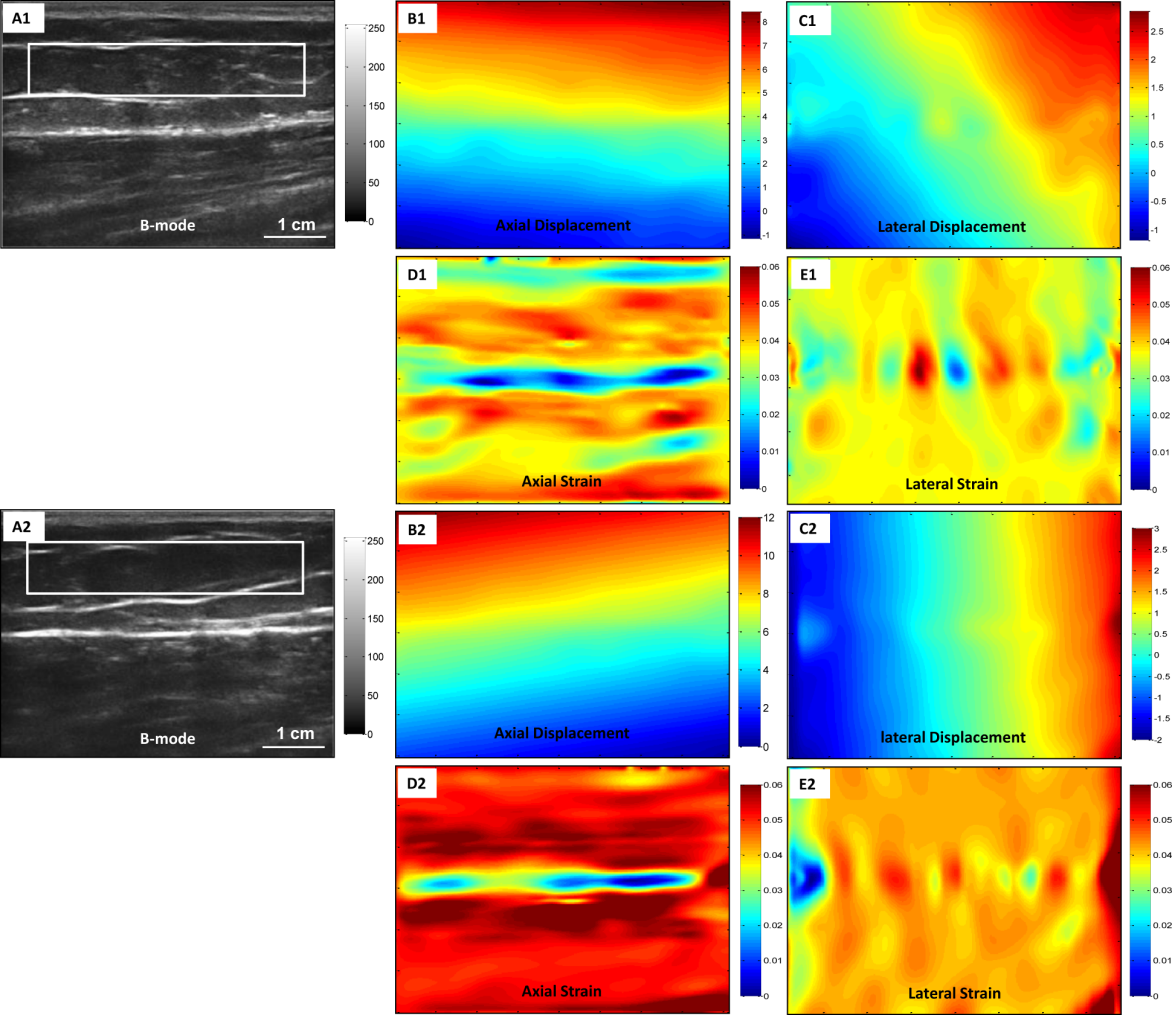
The *in-vivo* patient experimental results (right arm without lymphedema, and left arm with lymphedema). (A1) and (A2) are right and left arm B-mode images of a 50-year-old breast cancer patient with moderate degree lymphedema, 1 year post-radiotherapy. (B1) and (B2) are right and left axial displacement images. (C1) and (C2) are right and left lateral displacement images. (D1) and (D2) are right and left axial strain images. (E1) and (E2) are right and left lateral strain images.

**Fig 11:**
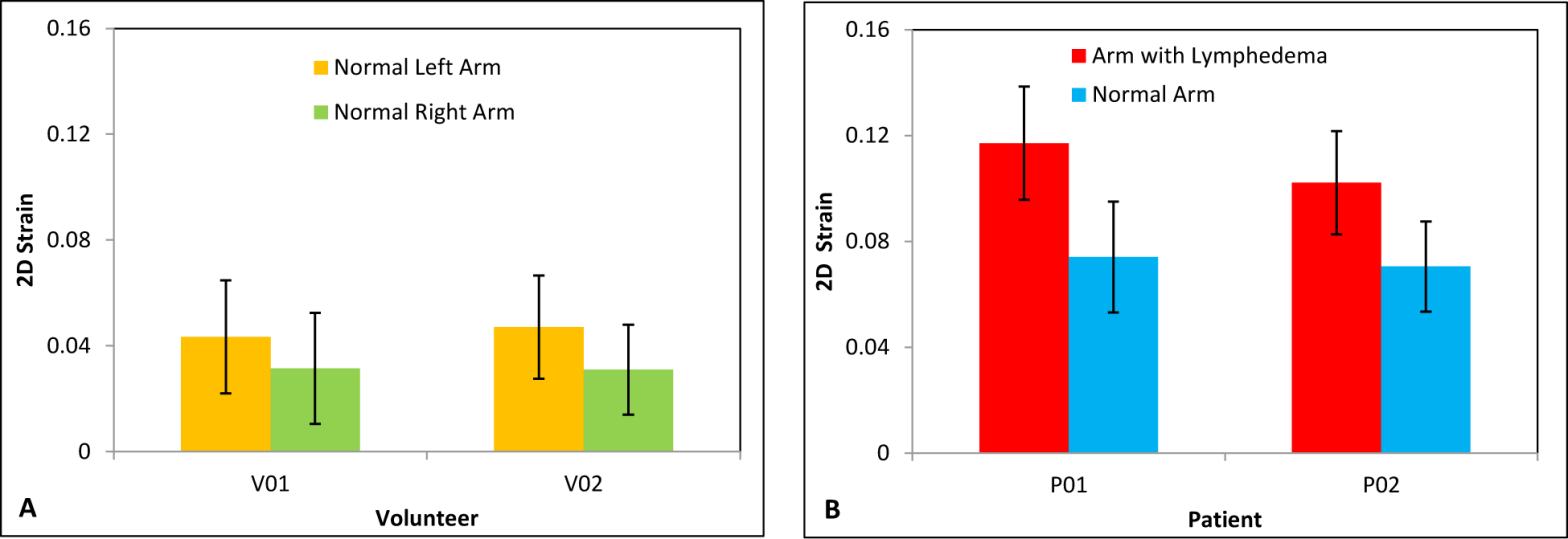
Average 2D strain comparison of two volunteers (A) and two patients (B).

Fig 10 shows the strain images of the control and the lymphedema-affected arms for a 50-year-old patient who was diagnosed with moderate (grade 2) lymphedema of the left arm. The strain images of the lymphedema-affected arms appear redder (higher value) than the two normal arms, which indicates that the arms with lymphedema appear softer than the control arms. To quantitatively evaluate the strain of arms, we calculated the averaged strain values inside the ROIs. Fig 11 displays a 2D strain comparison of the two volunteers and two patients. The average strain value of the lymphedema-affected arms was 1.5 times higher than those of the normal arms. The averaged strain value of arms with lymphedema is 2.8 times higher than those of two volunteers.

## Discussion

We have developed a registration-based displacement estimation approach for ultrasonic strain imaging. It is an alternative approach towards 2D strain estimation based on non-rigid registration of the US images. The novelty of our approach is in the similarity measures of the deformable registration and its application in 2D strain imaging. In this approach, a combined affine and B-spline transformation model is used to represent the displacement of tissue between pre-compression and post-compression B-mode image sequences. The 2D displacement between the different image frames is optimized by maximizing the NMI and minimizing NSSD of corresponding voxel intensities and region structure, which is a statistical intensity- and structure-based similarity measure that is not sensitive to the local intensity and contrast changes between the pre- and post-compression tissue. The 2D strain is computed from the displacement by spatial differentiation. In contrast to the traditional speckle tracking methods, no regularizing post-processing step is required, as regularization of the displacement is embedded in the optimization through the B-spline interpolation and the penalty. Our method does not need an implicit tissue property assumption and any post-processing because the displacement field is continuous, is not constrained to any particular set of directions and can measure 2D strain. We have demonstrated the feasibility and reliability through the elastography phantom and *in-vivo* clinical study.

Although many studies have used registration methods for elastography, to the best of our knowledge, we are the first to apply a hybrid non-rigid registration to estimate the 2D ultrasound strain images of lymphedema tissue. A number of methods have been proposed to estimate 2D or 3D strain based on image registration [34, 42–49]. Different registration-based approaches have been investigated for strain imaging, such as the optical flow, elastic registration between subsequent image frames or diffeomorphic registration. Richards *et*. *al*. applied a non-rigid registration based on a bi-linear, finite element-based displacement field to visualize the radial and circumferential component of strain within vascular tissue [42]. Liang *et*. *al*. employed an image registration based technique for intravascular ultrasound B-mode images of arteries [50]. Their technique used a free-form deformation model based on cubic B-splines, and only an intensity-based image similarity metric. Elen *et*. *al*. used an intensity-based spatio-temporal elastic registration of ultrasound images to measure cardiac strain tensor [51]. Chandrashekara *et*. *al*. presented a method based on nonrigid image registration using multilevel free-form deformations based on NMI for the analysis of myocardial strain using tagged MRI [52]. Ledesma-Carbayo *et*. *al*. used a semi-local spatio-temporal parametric model for deformation using splines to estimate the ultrasound cardiac strain [53]. De Craene *et*. *al*. presented a diffeomorphic registration algorithm, and applied it estimate motion and strain from 3D echocardiography images [44]. This approach enforced time consistency by representing the velocity field as the sum of continuous spatiotemporal B-Spline kernels. The sum of squared differences between the intensities of each frame and a reference one was used as the image similarity metric. Piella *et*. *al*. used a multi-view diffeomorphic registration strategy to estimate the transformation from the input multiple views rather than from a single view or a reconstructed compounded sequence [43]. Almost all of previously published registration-based methods were used to estimate the strain in the intravascular and cardiac US images. Although those methods worked well in intravascular and cardiac US images, they may not be suitable for the evaluation of lymphedematous tissues, which present completely different challenges. Lymphedema could affect various tissues including cutaneous, subcutaneous, fat, and possibly muscle layers. On the US images, the distinguishing layers are shown as high-contrast interfaces, which affect the performance of many CC-based strain measurements. However, the structure-based NSSD in our similarity measure uses the high-contrast interfaces between the tissue layers to calculate the displacement. Therefore, one of the strengths of the proposed registration-based method is to resolve the high-contrast interfaces.

The proposed registration-based strain method has the same solution as the B-mode images. Unlike the CC-based method, there is no trade-offs between resolution and noise level in this registration-based approach. In the CC-based method, the selection of the window length is a trade-off between the attainable resolution and noise level within the strain image [54]. Many methods have already been developed to improve this problem, such as using the filtering [55], multiscale estimation [23] and multi-compression average [56]. However, noise always is a challenging problem for the CC-based block matching method, especially due to the nonuniformity of the ultrasound field and non-rigid tissue deformation [24, 55]. In this study, a high SNR in the lateral direction was demonstrated in phantom study (Fig 8). One of a critical clinical need is early detection, because early detection leads to more successful intervention. Therefore, we focus on SNR and CNR because early detection means a technology to detect subtle changes. The SNR increased with pressure, which is consistent with the previous results that relatively large external deformations increase the SNR of displacement and strain images [23, 51, 56]. However, large deformations of soft tissues cannot be described with a linear elastic model [56–58]. In our phantom study, the strain curves in Fig 9 shows a nonlinear elastic model, which is also consistent with the previous study [58]. In this two-tissue (hard tissue enclosed by soft tissue) elastography phantom, the elastic modulus (stress/strain = λ) of the lesion remains constant (λ) at low stress and the lesion can be described as a linear elastic model. As the stress increases, the strain quickly increases and the elastic modulus (stress/strain<λ) of the lesion decreases as a function of strain (*i*.*e*., strain softening for the relatively hard lesion). The lesion cannot be described with a linear elastic model and, in our case, the increased 2D strain curve closely conformed to an increment exponential curve.

The proposed registration-based strain method uses global affine and local B-spines-based nonrigid transformations and copes well with large deformation. We identified several markers in ultrasound images to estimate the average deformation in axial and lateral directions. The average deformations at axial and lateral directions were up to 4.1% and 0.6% for the elastography phantom study, and 11.6% and 3.1% for the *in-vivo* patient study. Skovoroda *et al* applied their proposed method to reconstruct the strain image up to 16% axial deformation [58]. Deprez *et al* used a deformable model to the tissues and locally computed axial land lateral strains [59]. The method was reported to provide estimate up to 14% deformation for simulated data.

Recently, poroelastography has been developed to image the effective Poisson’s ratio (EPR) distribution in poroelastic materials under compression and its temporal behavior due to fluid flow [60, 61]. An EPR time constant (EPR-TC) elastogram can be generated from the time constant of the temporal decay of each pixel in the poroelastogram, and therefore may convey information about the underlying permeability distribution of the poroelastic material [62]. Righetti *et al*. applied a CC-based elastography based on poroelastic tissue model to estimate the strain of normal and lymphedematous tissue [63]. Even though the preliminary study showed early success in differentiating lymphedematous tissues in vivo, the authors pointed out high SNR and that only axial strain images were calculated. Even though this poroelastic tissue model might seem an attractive alternative, they are usually difficult to evaluate and verify. Moreover, the poroelastic properties of the lymphedema tissues may vary significantly across subjects and with age, which renders the application of such models difficult. In contrast to the poroelastic tissue model, the registration-based algorithm makes no assumptions about the poroelastic properties of the lymphedema tissue.

One of the limitations of this study is the small number of participants. We are conducting a clinical study with a large cohort to investigate EPR-TC curve using our method. We will further investigate the sensitivity of our method with a large cohort, and validate if our method could detect lymphedema at the very early stage and distinguish different stages of lymphedema well. All the experiments were conducted on an Intel Xeon 2.66-GHz CPU computer with MATLAB implementation.

## Conclusion

Lymphedema is a frequent complication of breast cancer and its therapies, and can have long-term physical and psychosocial consequences for patients. We have proposed a novel registration-based method to estimate the 2D strain of lymphedema. The phantom study showed that tissue strains could be recovered with accuracy and regions with deformation could be determined from these estimated strains. For the *in-vivo* study strain difference is shown between the normal and lymphedema-affected arms. This 2D ultrasound strain imaging tool may be useful as we try to obtain information regarding the potential effectiveness of lymphatic treatments and monitor the progress of therapy or test the efficacy of new treatments.
